# Downregulation of the cancer susceptibility protein WRAP53*β* in epithelial ovarian cancer leads to defective DNA repair and poor clinical outcome

**DOI:** 10.1038/cddis.2015.250

**Published:** 2015-10-01

**Authors:** E Hedström, C Pederiva, J Farnebo, B Nodin, K Jirström, D J Brennan, M Farnebo

**Affiliations:** 1 Department of Oncology-Pathology, Cancer Centrum Karolinska, Karolinska Institutet, Stockholm, Sweden; 2 Department of Diagnostic Radiology, Karolinska University Hospital and Department of Molecular Medicine and Surgery, Karolinska Institutet, Stockholm, Sweden; 3 Division of Oncology and Pathology, Department of Clinical Sciences, Lund University, Lund, Sweden; 4 Queensland Centre for Gynecological Cancer, University of Queensland, School of Medicine, Central Clinical Division, Brisbane, Australia

## Abstract

Alterations in the scaffold protein WRAP53*β* have previously been linked to carcinogenesis and, in particular, associated with an increased risk for epithelial ovarian cancer. Here, we investigated the pathogenic impact and prognostic significance of WRAP53*β* in connection with epithelial ovarian cancer and examined the underlying mechanisms. We find that reduced expression of WRAP53*β* in ovarian tumors correlated with attenuated DNA damage response and poor patient survival. Furthermore, in ovarian cancer cell lines, WRAP53*β* was rapidly recruited to DNA double-strand breaks, where it orchestrated the recruitment of repair factors involved in homologous recombination and non-homologous end joining, including RNF168, 53BP1, BRCA1 and RAD51. Mechanistically, WRAP53*β* accomplishes this by facilitating the necessary ubiquitinylation at DNA breaks. Finally, we demonstrate that loss of WRAP53*β* significantly impairs the repair of DNA double-strand breaks, resulting in their accumulation. Our findings establish WRAP53*β* as a regulator of homologous recombination and non-homologous end joining repair in ovarian cancer cells, suggesting that loss of this protein contributes to the development and/or progression of ovarian tumors. Moreover, our current observations identify the nuclear levels of WRAP53*β* as a promising biomarker for the survival of patients with ovarian cancer.

Accounting for 2% of all cancers in women, but ranking fifth among the causes of all cancer-related deaths in women, ovarian cancer is associated with the highest mortality rate among gynecological malignancies.^1^ Its poor prognosis is primarily due to late diagnosis, since the symptoms do not usually appear until the disease has spread outside the ovaries. Most ovarian cancers are epithelial and treatment usually includes cytoreductive surgery (debulking) followed by chemotherapy (platinum-based drugs). Unfortunately, the majority of patients who respond to primary chemotherapy later experience relapse.

Alteration of the DNA damage response is one major factor in the onset and/or progression of ovarian cancer. For example, repair by homologous recombination (HR) is defective in approximately half of all ovarian tumors due to inactivation of genes encoding proteins involved in this pathway, such as *BRCA1* and *BRCA2*.^2^ Since HR is involved in repairing DNA lesions caused by platinum-based chemotherapeutics and poly ADP-ribose polymerase (PARP) inhibitors, HR-deficient tumors are hypersensitive to these drugs, which help prolong patient survival,^3, 4^ although perhaps for not more than 5 years.^5, 6, 7^

When DNA double-strand breaks arise, the high-fidelity HR pathway and error-prone non-homologous end joining (NHEJ) pathway compete to repair them. Inactivation of HR enhances NHEJ repair and overstimulation of this error-prone pathway was recently shown to contribute to the hypersensitivity of HR-deficient ovarian tumors to PARP inhibitors.^8, 9^ Consistent with this observation, inactivation of NHEJ through inhibition of DNA-PK/Ku80 or deletion of 53BP1 abrogates the cytotoxicity and genomic instability induced by PARP inhibitors leading to drug resistance.^8, 9^ Thus, impairment of NHEJ in HR-deficient tumors could result in resistance to treatment and reduce patient survival.

Previously, we identified the gene *WRAP53* (WD40-encoding RNA antisense to p53) and showed that its product (referred to as WRAP53*α*) regulates expression and function of the tumor suppressor p53.^10^ *WRAP53* also encodes a scaffold protein designated WRAP53*β* (alias WRAP53, WDR79 or TCAB1), which is present both in the cytoplasm and nucleus, where it is highly enriched in the nuclear organelles Cajal bodies. WRAP53*β* clearly plays a central role in the maintenance and localization of factors involved in splicing and telomere elongation to the Cajal body^11, 12, 13^ and was also recently shown to control the repair of DNA double-strand breaks by both the HR and NHEJ pathways through targeting the critical ubiquitin ligase RNF8 to these lesions.^14^

Loss of WRAP53*β* function is associated with various disorders, including dyskeratosis congenita, which is caused by germline mutations in WRAP53*β* and characterized by bone marrow failure and predisposition for cancer^15^ and spinal muscular atrophy, a neurodegenerative disorder that is the leading genetic reason of infant mortality worldwide.^13^ Moreover, single nucleotide polymorphisms in *WRAP53* or altered expression of the protein are correlated with an elevated risk of developing a variety of sporadic tumors, including ovarian, breast, head and neck cancers.^16, 17, 18, 19, 20^ Nonetheless, the exact involvement of WRAP53*β* in carcinogenesis remains unclear.

In the current investigation, we find that attenuated expression of WRAP53*β* contributes to the progression of and is associated with altered DNA damage response in epithelial ovarian cancer. In this context, we demonstrate that WRAP53*β* participates in DNA repair in epithelial ovarian cancer cell lines by targeting factors involved in the HR and NHEJ pathways to such DNA lesions and that loss of this protein eliminates repair of DNA double-strand breaks. In summary, we establish a distinct role for this protein in the DNA damage response and repair in ovarian cancer cells and propose that WRAP53*β* thereby acts as a tumor suppressor in connection with epithelial ovarian cancer.

## Results

### Reduced levels of WRAP53*β* mRNA and protein correlate with shorter survival in ovarian cancer patients

**Figure 1:**
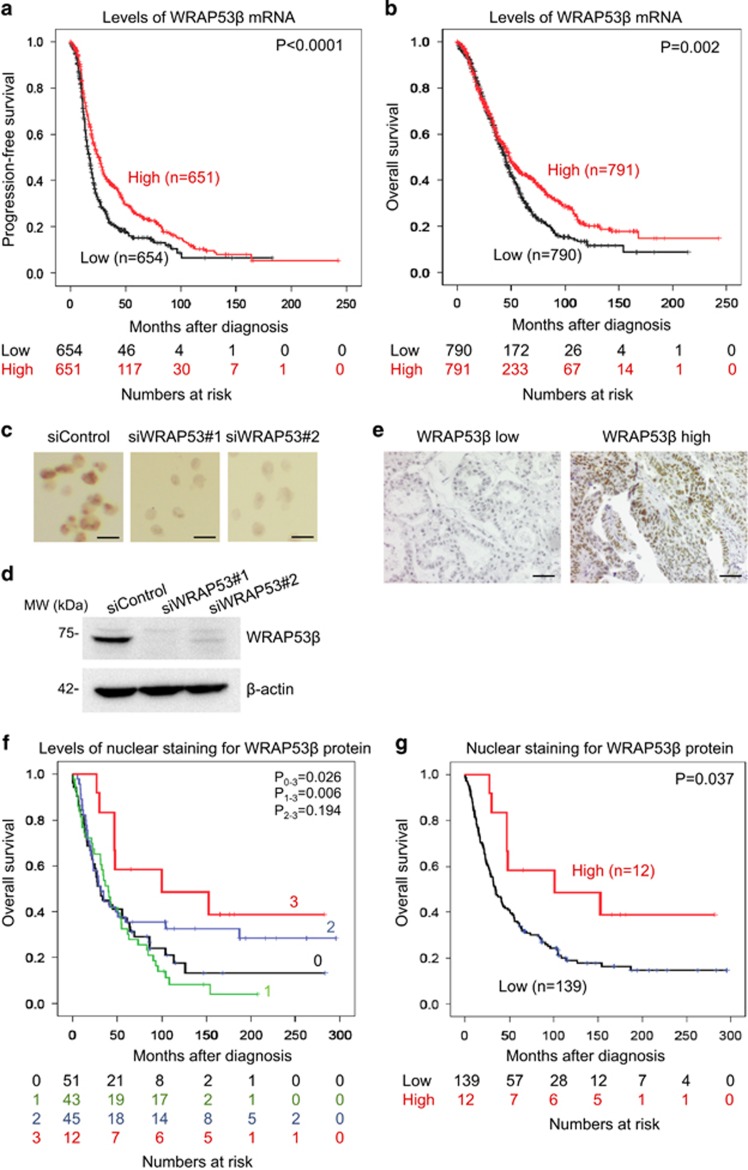
Relationship between the levels of WRAP53*β* and survival of patients with ovarian cancer. (**a**) Kaplan-Meier analysis of progression-free and (**b**) overall survival of patients with epithelial ovarian cancer in relation to the level of WRAP53*β* mRNA. (**c**) Immunohistochemical analysis of formalin-fixed and paraffin-embedded U2OS cells transfected with indicated siRNA oligonucleotides for 48 h. Scale bars, 20 *μ*m. (**d**) Western blotting of WRAP53*β* and *β*-actin in U2OS cells treated with the indicated siRNA oligonucleotides for 48 h. (**e**) Immunohistochemical staining of tumors expressing low and high levels of WRAP53*β*. Scale bars, 50 *μ*m. (**f**) Kaplan-Meier analysis of patient survival in relation to the degree of immunohistochemical staining for WRAP53*β* in the nucleus. Scoring: 0=negative (*n*=51), 1=weak (*n*=43), 2=moderate (*n*=45) and 3=strong (*n*=12). (**g**) Kaplan-Meier analysis of the same data as in (**f**) after grouping of the tumors into those expressing low (0–2) and high (3) nuclear levels of WRAP53*β*

Kaplan-Meier analysis of WRAP53*β* mRNA levels in epithelial ovarian cancer cohort I revealed that lower levels were associated with shortened progression-free and overall survival (Figures 1a and b). After confirming the specificity of the WRAP53*β* antibody by immunohistochemistry and western blotting of tumor cells either expressing or lacking this protein (Figures 1c and d) immunohistochemical analysis of tumor samples (cohort II) revealed tumor-specific and nuclear expression of WRAP53*β* of varying degrees (Figure 1e). Kaplan-Meier analysis of the nuclear levels revealed that lower intensity of nuclear staining for WRAP53*β* was correlated with reduced survival of the patients with ovarian cancer (Figure 1f). For statistical comparison, the tumors were grouped into those exhibiting low (combined score 0–2) and high (score 3) nuclear staining for WRAP53*β* (Figure 1g).

Examination of the relationship between nuclear expression of WRAP53*β* and clinical variables revealed significant correlations between the levels of expression and stage (*P*=0.009) and differentiation grade (*P*=0.049) of the tumors (Table 1), but not with age, histology or levels of p53 (data not shown). Multivariate analysis including nuclear expression of WRAP53*β* and stage, differentiation grade, histology and p53 expression of the tumors revealed that low WRAP53*β* expression was associated with a 4-fold higher risk of dying from ovarian cancer and demonstrated that WRAP53*β* is an independent marker of survival in patients with ovarian cancer (HR=4.20, 95% CI=1.00–17.61, *P*=0.05; Table 2).

Together, these findings suggest that nuclear expression of WRAP53*β* correlates with the progression of epithelial ovarian cancer and might serve as a prognostic marker for this type of tumor.

### WRAP53*β* and the DNA damage response show a positive correlation in ovarian tumors

**Figure 2:**
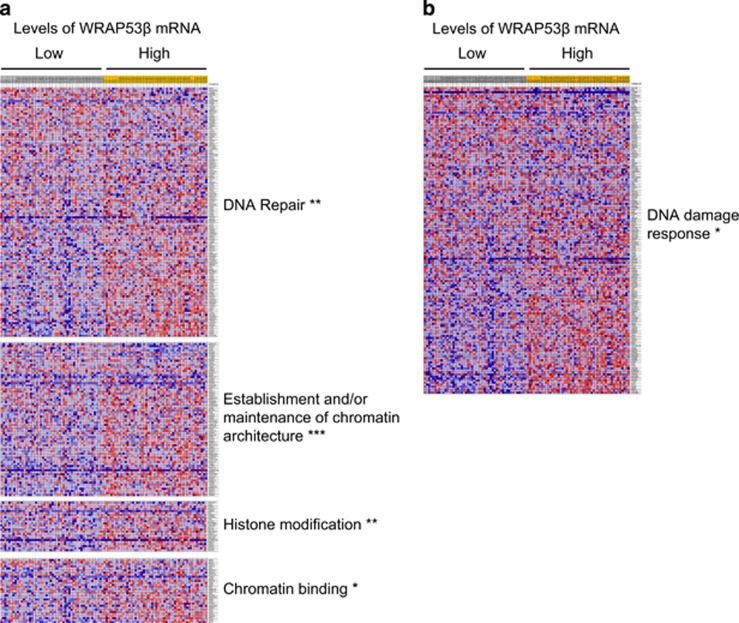
The levels of mRNA for WRAP53*β* and processes involved in the DNA damage response show a positive correlation in ovarian tumors. Heat map of mRNAs encoding proteins involved in the DNA damage response for tumors expressing highest and lowest levels of WRAP53*β* mRNA. (**a**) Gene set enrichment analysis of cohort III demonstrated that higher levels of WRAP53*β* mRNA were strongly associated with higher levels of mRNAs encoding factors involved in DNA repair, chromatin architecture, histone modification and chromatin binding (*P<0.05, **P<0.01, ***P<0.001 and false discovery rate <0.25). (**b**) Gene set enrichment analysis of cohort IV demonstrated that higher levels of WRAP53*β* mRNAs were strongly associated with higher levels of mRNAs encoding factors involved in the DNA damage response (*P<0.05, false discovery rate <0.25). Genes were ranked on the basis of their signal-to-noise ratios. Expression values, also based on the signal-to-noise ratios, are color coded: red=high, pink=moderate, light blue=low and dark blue=lowest

To gain a deeper understanding of the underlying molecular processes associated with WRAP53*β* expression in epithelial ovarian cancer, we performed gene set enrichment analysis (GSEA) of two independent cohorts, both of which are included in the Kaplan-Meier analysis presented in Figures 1a and b. This showed in cohort III an association between high levels of WRAP53*β* and of a number of processes involved in the DNA damage response, including DNA repair, chromatin architecture, histone modification and histone binding (*n*=241; Figure 2a). GSEA of cohort IV (*n*=403) confirmed this association (Figure 2b). Together, these observations suggest that WRAP53*β* plays an important role in the DNA damage response in epithelial ovarian cancer and that attenuation of this function may contribute to tumor formation, progression and therapeutic response.

### WRAP53*β* regulates repair of DNA double-strand breaks in ovarian cancer cell lines

**Figure 3:**
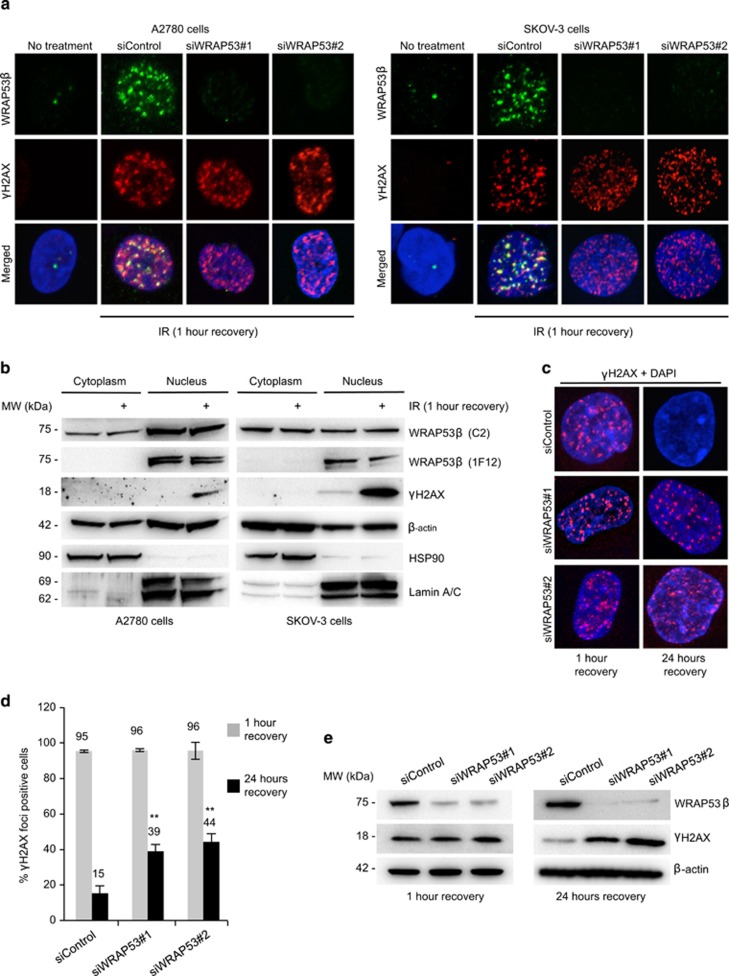
WRAP53*β* accumulates at the sites of DNA damage and promotes DNA repair in the ovarian cancer cell lines A2780 and SKOV-3. (**a**) A2780 and SKOV-3 cells were treated with siControl or two different WRAP53*β*-targeting oligonucleotides (siWRAP53#1 and siWRAP53#2) for 48 h, irradiated (6 Gy, 1-h recovery), fixed after preextraction with CSK buffer and immunostained for WRAP53*β* and *γ*H2AX. (**b**) Western blot analysis of cytoplasmic and nuclear fractions from untreated or irradiated (6 Gy, 1-h recovery) A2780 and SKOV-3 cells. In all fractions HSP90 and lamin A/C were used as cytoplasmic and nuclear markers, respectively. (**c**) A2780 cells were treated with the same siRNAs as in (**a**) for 24 h; exposed to 6 Gy IR, fixed for 1 h or for 24 h later and immunostained for *γ*H2AX. (**d**) Quantification of the results in (**c**), showing the percentage of nuclei containing >10 *γ*H2AX foci (*n*=200). The error bars depict the S.E.M.; *n*=3, **P<0.01, as determined by Student's *t*-test. (**e**) A2780 cells were treated as in (**c**) and then subjected to western blotting for WRAP53*β*, *γ*H2AX and *β*-actin

To further explore whether WRAP53*β* is involved in DNA repair of ovarian tumors, we studied the behavior of this protein following DNA damage in the ovarian cancer cell lines A2780 and SKOV-3. One hallmark of DNA repair proteins is their accumulation at the sites of damage, often forming discrete foci. Following exposure of the ovarian cancer cell lines A2780 and SKOV-3 to ionizing radiation (IR), WRAP53*β* was rapidly recruited to sites of DNA damage in these cells (Figure 3a). Moreover, the IR-induced foci formed by WRAP53*β* clearly overlapped with the foci containing Ser139-phosphorylated histone H2AX (referred to as *γ*H2AX), a marker of DNA damage. Furthermore, the WRAP53*β* staining was specific, since it was abolished by siRNA knockdown of WRAP53*β* (Figure 3a). Thus, WRAP53*β* is recruited to sites of DNA damage in ovarian cancer cell lines. The possibility that the intracellular distribution of WRAP53*β*, which is located both in the nucleus and cytoplasm,^13^ is altered by irradiation was examined using A2780 and SKOV-3 cells. The rabbit *α*-WRAP53-C2 antibody, which detects both the cytoplasmic and nuclear forms, was used, since the mouse *α*-WDR79 clone 1F12 antibody only recognizes nuclear WRAP53*β*. The lack of change in intracellular distribution upon irradiation (Figure 3b) indicates that the WRAP53*β* protein recruited to the sites of DNA damage originates from the nuclear pool.

This recruitment of WRAP53*β* to DNA breaks indicates its direct involvement in DNA repair, and, indeed in control cells expressing WRAP53*β*, the majority of *γ*H2AX foci rapidly formed in response to IR was resolved 24 h later reflecting efficient DNA repair (Figures 3c and d). In contrast, in cells depleted of WRAP53*β* recovery from DNA damage was severely delayed and a significant number of *γ*H2AX foci remained even 24 h after IR. Western blotting confirmed that the level of *γ*H2AX in these WRAP53*β*-deficient cells remained elevated 24 h post IR (Figure 3e). Together, these findings demonstrate that WRAP53*β* is directly involved in the repair of DNA double-strand breaks in ovarian cancer cells.

### Knockdown of WRAP53*β* impairs recruitment of DNA repair factors to DNA breaks in ovarian cancer cell lines

**Figure 4:**
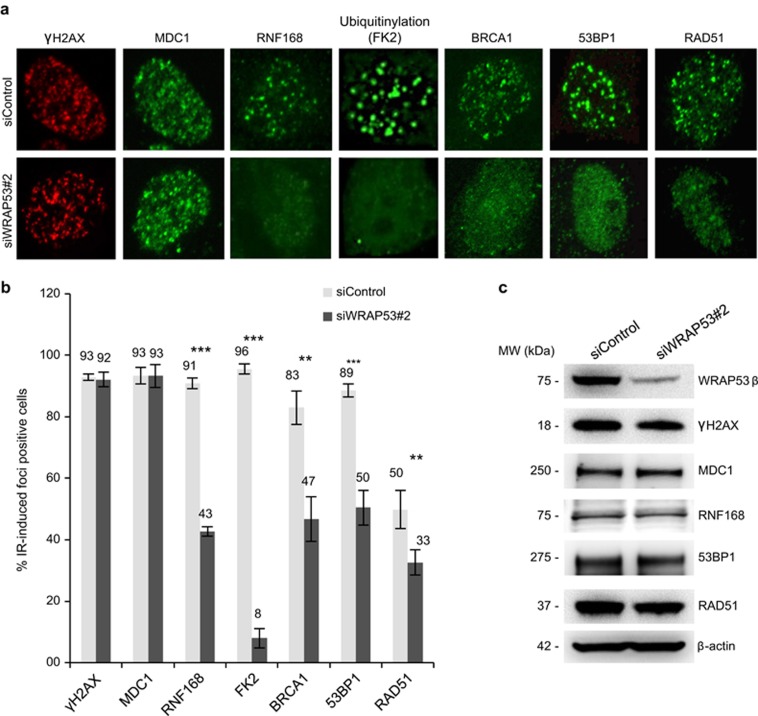
WRAP53*β* plays an important role in recruitment of factors involved in HR and NHEJ to DNA breaks in A2780 cells. (**a**) A2780 cells were transfected with siControl or siWRAP53#2 oligonucleotides for 48 h; exposed to IR (6 Gy), and 1 h later immunostained for *γ*H2AX, MDC1, RNF168, FK2 (recognizes conjugated ubiquitin), BRCA1, 53BP1 and RAD51. (**b**) Quantification of the results in (**a**), as the percentage of 200 cells counted in each experiment whose nuclei contained >10 IR-induced foci. The error bars depict the S.E.M.; *n*=3, **P<0.01 and ***P<0.001, as determined by Student's *t*-test. (**c**) A2780 cells were treated as in (**a**) and then subjected to western blotting for WRAP53*β*, *γ*H2AX, MDC1, RNF168, 53BP1, RAD51 and *β*-actin. We could not assess the protein levels of BRCA1 due to a lack of antibodies that work for western blotting

HR inactivation contributes to ovarian tumorigenesis, and we observed reduced accumulation of key factors involved in HR and NHEJ, including BRCA1 (HR), RAD51 (HR) and 53BP1 (NHEJ) at DNA double-strand breaks in irradiated A2780 cells depleted of WRAP53*β*. At the same time, the upstream proteins *γ*H2AX and MDC1 still formed foci (Figures 4a and b). Thus, loss of WRAP53*β* leads to defective accumulation of critical factors mediating HR and NHEJ to sites of DNA damage in ovarian cancer cells.

To explore the underlying mechanism, we monitored ubiquitinylation at the sites of DNA damage known to be important for the local accumulation of BRCA1, 53BP1 and RAD51 but not *γ*H2AX and MDC1 at such site.^14, 21, 22, 23, 24^ For detection of ubiquitinylation at the sites of DNA damage, we used the FK2 antibody, which binds to ubiquitin chains on mono- and polyubiquitinated proteins, but not free ubiquitin. Indeed, knockdown of WRAP53*β* reduced the accumulation of both the E3 ligase RNF168, which catalyzes this ubiquitinylation, and conjugated ubiquitin at DNA double-strand breaks (Figures 4a and b). This impaired accumulation at DNA damage sites was not due to altered levels of these factors (Figure 4c). Altogether, our results demonstrate that WRAP53*β* regulates the repair of DNA double-strand breaks in ovarian cancer cells and that loss of this protein leads to defects in both HR and NHEJ.

## Discussion

Here, we demonstrate for the first time that low nuclear expression of the scaffolding protein WRAP53*β* correlates with aggressiveness and poor prognosis of epithelial ovarian cancer. A similar observation was recently reported, where loss of nuclear WRAP53*β* is associated with reduced survival and enhanced radioresistance in patients with head and neck cancer.^20^ This correlation was observed only for WRAP53*β* in the nucleus and not in the cytoplasm,^20^ emphasizing that the subcellular localization of this protein should be taken into account when predicting outcome. In addition, single nucleotide polymorphisms in the *WRAP53* gene are associated with poor outcome in epithelial ovarian cancer^18, 19^ and inherited mutations in this same gene cause dyskeratosis congenita, which is associated with a dramatic elevation in risk for developing cancer.^15^ Since inactivation of WRAP53*β* by mutations in both alleles is required for development of this disease, this protein appears to be a *bona fide* tumor suppressor.

Reductions in the levels of both WRAP53*β* mRNA and protein were associated with shorter survival in patients with epithelial ovarian cancer. This suggests that downregulation of WRAP53*β* in such tumors occurs at the transcriptional or post-transcriptional level rather than post-translationally, although this remains to be determined.

We have shown that ovarian tumors expressing low levels of WRAP53*β* exhibit downregulation of key factors involved in the DNA damage response, indicating impaired DNA repair. Indeed, in ovarian cancer cell lines WRAP53*β* rapidly accumulates at DNA breaks, where it orchestrates the accumulation of DNA repair proteins involved in both HR and NHEJ, including RNF168, 53BP1, BRCA1 and RAD51. WRAP53*β* achieves this recruitment by promoting ubiquitinylation at the sites of DNA damage, in agreement with our recent findings that WRAP53*β* serves as a scaffold for complex formation between the E3 ligase RNF8 and the anchoring protein MDC1.^14^ Accordingly, knockdown of WRAP53*β* impairs the repair of DNA double-strand breaks by both HR and NHEJ resulting in their accumulation. These observations suggest that attenuated expression of this protein contributes to genomic instability and carcinogenesis.

Our findings indicate that the WRAP53*β* recruited to DNA lesions originates from the nuclear pool alone. This may explain why lower levels of nuclear, but not of cytoplasmic WRAP53*β* are associated with poor prognosis and radioresistance in cases of head and neck cancer,^20^ as well as with altered DNA repair and poor prognosis in patients with ovarian cancer.

Precancerous lesions are characterized by activation of the DNA damage response (often due to replication stress), which is believed to eliminate hazardous cells. At an early stage in the development of cancer, this defense is lost by inactivation of factors involved in the DNA damage response, which contributes to progression to carcinoma.^25, 26^ At this early stage, the p53 protein is still active and promotes removal of dangerous cells through growth arrest or apoptosis. Subsequent inactivation of p53, often occurring at a later stage in tumor development, results in survival of damaged cells, which augment tumor progression and aggressiveness.

In line with this model, we find that the levels of WRAP53*β* are higher in ovarian cancer cells than nonmalignant tubal cells indicating activation of the DNA damage response in these cells. Our findings further demonstrate that subsequent downregulation of WRAP53*β* in ovarian cancer cells impairs their damage response and drives tumor progression. Moreover, patients whose tumors exhibited both a low level of nuclear WRAP53*β* and positive/high p53 expression, indicative of mutation, suffered a higher rate of mortality compared to those with both high-nuclear WRAP53*β* and no expression of p53 (HR=4.71, 95% CI=1.15–19.33, *P*=0.032). Although the mutational status of p53 needs to be verified by sequencing, these data indicate that inactivation of p53 in WRAP53*β*-deficient cells contributes further to tumor progression and aggressiveness.

Our own findings and those of others reveal that appropriate expression of p53 is dependent on WRAP53*α*, which also is encoded by the *WRAP53* gene, and, moreover, that p53 activity in response to DNA damage is abrogated when WRAP53*α* is downregulated.^10, 27^ Several lines of evidence indicate that WRAP53*α* and WRAP53*β* act independently and that neither WRAP53*β* transcripts nor protein are involved in regulating p53.^10, 28^ However, in tumors containing reduced levels of WRAP53*β* transcripts, such as ovarian cancer, WRAP53*α*, which is transcribed from the same locus, might also be downregulated resulting in inactivation of p53.

Still, the involvement of WRAP53*β* in the repair of DNA double-strand breaks is independent of WRAP53*α*-mediated regulation of p53, since this also occurs in SKOV-3, H1299 and HeLa cells, which contain no or inactive p53 (Figures 3 and 4).^14^ Nonetheless, it remains to be determined whether the parallel actions of WRAP53*α* and WRAP53*β* are required for a complete DNA damage response that protects against tumor development and/or progression.

We have also established that WRAP53*β* is an upstream regulator of BRCA1. Since these proteins act in the same pathway of DNA repair, inactivation of either of these proteins in a tumor may impair HR. Alternatively, downregulation of WRAP53*β* in *BRCA1/2*-mutated tumors might inactivate NHEJ and induce drug resistance. However, such questions remain to be examined.

A hallmark of *BRCA1/2*-mutated carcinomas is their hypersensitivity to platinum-based chemotherapy and PARP inhibitors. However, early studies have suggested that, for unknown reasons, only 30–40% of *BRCA1/2*-mutated ovarian and breast cancers respond to PARP inhibitors.^29, 30, 31^ The demonstration that functional NHEJ contributes to the cytotoxicity of such inhibitors suggests that HR-deficient cancers with diminished NHEJ will be relatively resistant. This line of reasoning might explain why loss of WRAP53*β*, which impairs both HR and NHEJ, shortens the survival of epithelial ovarian cancer patients. Further investigations on the contribution of WRAP53*β* to the response of ovarian cancer to treatment may reveal whether its downregulation leads to drug resistance, thereby helping to design individualized treatment.

In summary, our present findings indicate that nuclear levels of WRAP53*β* are a promising biomarker for prediction of the clinical outcome of epithelial ovarian cancer hopefully contributing to novel treatment strategies and improved survival. Moreover, our observations establish altered DNA repair as a cause of WRAP53*β*-associated ovarian cancer and suggest that defects in DNA repair may contribute to other forms of WRAP53*β*-related cancer as well.

## Materials and Methods

### Characterization of patients

#### WRAP53*β* mRNA

By using microarray data on overall and progression-free survival for 1581 patients (cohort I), WRAP53*β* expression was assessed using the KM-plotter meta-analysis software (2015 version; http://kmplot.com^32^) and the JetSet best probe (44563_at). Gene Expression Omnibus (GEO) IDs: GSE14764, GSE15622, GSE19829, GSE3149, GSE9891, GSE18520, GSE26712 and TCGA (The Cancer Genome Atlas). The median expression value was used as a threshold for survival analysis. Patients whose tumors exhibited WRAP53*β* mRNA levels above this threshold were classified as high expressers, and those with WRAP53*β* mRNA levels below this threshold was classified as low expressers.

#### WRAP53*β* protein

This analysis involved a composite of two prospective, population-based cohorts from the Malmö Diet and Cancer study (MDCS; *n*=101) and Malmö Preventive Project (MPP; *n*=108) with epithelial ovarian cancer tumors collected until 31 December 2007. Thirty-five patients participated in both studies and archived tumor tissue for 154 of the 174 cases could be retrieved, all but three of which were suitable for analysis (*n*=151, cohort II). Information on clinical stage was obtained from medical charts and histopathological evaluations from pathology records. The tumors were divided into four groups on the basis of histological subtype: serous (*n*=90), endometrioid (*n*=35), mucinous (*n*=12) and others (*n*=17). The latter group included clear cell (*n*=9), Brenner (*n*=1) and unknown (*n*=7) tumors. The median age at the time of diagnosis was 62 (range 47–83) years. Information on the cause of death before 30 June 2012 in the cases of epithelial ovarian cancer was retrieved from medical charts and the Swedish Cause-of-Death Registry. Follow-up began at the time of diagnosis and ended with death, emigration or on 30 June 2012, whichever occurs first. Following a median follow-up of 3.00 years (range 0–24.63), 122 patients (79.2%) were dead, 112 of these (72.3%) from ovarian cancer and 32 (20.8%) were still alive. The study cohort involved here has been described in detail previously.^33, 34, 35^

### Statistical analysis

Kaplan-Meier analysis and the log-rank test were applied to relate overall and progression-free survival to WRAP53*β* expression. Pearson's chi-square test was used to explore potential associations between WRAP53*β* expression and clinicopathological parameters. Both uni- and multivariable Cox regression analysis were used to estimate hazard ratios for death from ovarian cancer in relationship to WRAP53*β* expression, with adjustment for the stage, differentiation grade, histology and p53 levels of the tumors. All calculations were performed using the SPSS version 19.0 software (SPSS Inc, Chicago, IL, USA) and *P*-values <0.05 were considered statistically significant.

### Gene set enrichment analysis

Gene set enrichment was analyzed using GSEA software (http://www.broadinstitute.org/gsea/index.jsp) as described previously.^36^ In these analyses, additional cohorts (III and IV) were used, both of which are included in cohort I. Cohort III originally consisted of 285 cases of epithelial ovarian cancer, fallopian tube and primary peritoneal cancers, as described previously.^37^ In the present case, patients with potential tumors of low malignancy and those who received neoadjuvant chemotherapy were excluded, leaving a final total of 241 cases.

Cohort IV consisted of 566 patients with high-grade serous ovarian cancer characterized in connection with TCGA project described previously.^2^ The present analysis was restricted to 403 of these samples profiled on the Affymetrix U133A platform. Expression data were downloaded from the GEO website (http://www.ncbi.nlm.nih.gov/gds/), GEO (http://www.ncbi.nlm.nih.gov/geo) or the TCGA data portal (https://tcga-data.nci.nih.gov/tcga/tcga-Home2.jsp). The R package ‘Affy' (http://www.bioconductor.org) was used to normalize the CEL files with the RMA procedure.^38^

For WRAP53*β*, normalized gene expression values were extracted from each data set and used without further modification. From each cohort III and IV, the 50 tumors expressing the highest levels of WRAP53*β* mRNA and the 50 tumors not expressing or expressing the lowest levels of WRAP53*β* mRNA were selected for comparison by GSEA. Arrays were compared on the basis of the signal-to-noise ratio using the gene set C.5 (all) v 2.5.

### Immunohistochemical staining

Tissue microarrays were constructed as described previously.^33, 34^ Heat-mediated antigen retrieval (pH=9) was performed with the PT-link system and immunohistochemical staining in the DAKO Autostainer system (Dako, Glostrup, Denmark) using *α*-WRAP53 (1 : 25 dilution, # HPA023026, Atlas Antibodies, Stockholm, Sweden) and *α*-p53 (1 : 200 dilution, #AMAb90956, Atlas Antibodies). Normal matched fallopian tube samples with no evidence of histological disease (*n*=39) were stained as negative controls. Staining intensity of WRAP53*β* was assessed by two of the authors as 0=negative, 1=weak, 2=moderate or 3=strong. For statistical purposes, the staining scores were subdivided into low (0–2, *n*=139) and high (3, *n*=12). Staining intensity of p53 was also assessed by two of the authors as positive or negative.

### Cells and culture conditions

Epithelial ovarian cancer cell lines A2780 (from chemonaive primary tumor) and SKOV-3 (from ascites fluid) were maintained in RPMI (HyClone, Thermo Scientific, Stockholm, Sweden), supplemented with 10% fetal bovine serum (HyClone) and 2.5 *μ*g/mL Plasmocin (InvivoGen, Toulouse, France) at 37 °C under 5% CO_2_ in humidified incubators. The identities of both cell lines were validated during 2012 using short-tandem repeat analysis AmpFlSTR Identifiler kit (Applied Biosystems/Life Technologies, Stockholm, Sweden).^39^

### siRNA transfections

#### siRNA, 10 nM

siWRAP53#1 (cat. no. SI00388941, Qiagen, Sollentuna, Sweden), siWRAP53#2 (cat. no. SI00388948, Qiagen) or siControl (cat. no. 1027280, Qiagen) was transfected into cells using HiPerFect (Qiagen) transfection reagent in accordance with the supplier's recommendations.

### Antibodies

#### Primary

Rabbit *α*-WRAP53-C2 (cat. no. PA-2020-100, Innovagen AB, Lund, Sweden, used for western blotting), mouse monoclonal *α*-WDR79 (clone 1F12, cat. no. H00055135-M04, Abnova (VWR International, Stockholm, Sweden), used for immunofluorescence), rabbit *α*-WRAP53 (cat. no. HPA023026, Atlas Antibodies, used for immunohistochemistry), mouse *α*-*γ*H2AX (cat. no. 05-636, Millipore, Solna, Sweden), rabbit *α*-*γ*H2AX (cat. no. 2577, Cell Signaling, Bionordika, Stockholm, Sweden), rabbit *α*-MDC1 (cat. no. ab11169, Abcam, Cambridge, UK), rabbit *α*-RNF168 (cat. no. ABE367, Millipore), mouse *α*-conjugated ubiquitin (FK2; cat. no. ST1200, Calbiochem, Millipore), rabbit *α*-53BP1 (cat. no. NB100-904, Novus Biologicals, Bio-Techne, Abingdon, UK), mouse *α*-BRCA1 (cat. no. sc-6954, Santa Cruz Biotechnology, Heidelberg, Germany), rabbit *α*-RAD51 (cat. no. sc-8349, Santa Cruz Biotechnology), mouse *α*-*β*-actin (Sigma-Aldrich, Stockholm, Sweden), mouse HSP90 *α*/*β* (cat. no. sc-13119, Santa Cruz Biotechnology) and rabbit lamin A/C (cat. no. sc-20681, Santa Cruz Biotechnology) were all used in this study.

#### Secondary

Goat *α*-rabbit HRP (cat. no. 7074, Cell Signaling), horse *α*-mouse HRP (cat. no. 7076, Cell Signaling), goat *α*-rabbit Alexa Fluor 488 (cat. no. A11008, Invitrogen, Stockholm, Sweden), goat *α*-mouse Alexa Fluor 488 (cat. no. A11029, Invitrogen), donkey *α*-rabbit Alexa Flour 594 (cat. no. A21207, Invitrogen) and donkey *α*-mouse Alexa Fluor 594(cat. no. A21203, Invitrogen) were all used in this study.

### Immunofluorescence microscopy

Cells grown on sterilized coverslips were fixed with 4% paraformaldehyde for 15 min at room temperature. They were then permeabilized with 0.1% Triton X-100 (Sigma-Aldrich) for 5 min at room temperature, followed by 30 min of blocking in blocking buffer (2% BSA, 5% glycerol, 0.2% Tween-20 and 0.1% NaN_3_). The coverslips were subsequently incubated for 1 h in primary antibody followed by 40 min in secondary antibody, both diluted in blocking buffer, and finally mounted with VECTASHIELD mounting medium containing DAPI (4′,6-diamidino-2-phenylindole, Vector Laboratories, Bionordika, Stockholm, Sweden). Images were acquired with a Zeiss Axioplan 2 microscope (Zeiss, Stockholm, Sweden) equipped with an AxioCam HRm camera (Zeiss) using 40 or 63 oil immersion lenses and processed using AxioVision Release 4.7 or with a LSM700 confocal microscope (Zeiss), mounted on Zeiss Axio observer.Z1 equipped with Plan-Apochromat 63x/1.4 oil immersion lenses, and processed using Zen 2012 Black.

#### Preextraction

To visualize IR-induced foci formed by WRAP53*β* and MDC1, the cells were first washed with PBS and then incubated for 3 min at room temperature with cytoskeleton buffer (CSK; 10 mM pipes (pH 7.0), 100 mM NaCl, 300 mM sucrose, 3 mM MgCl_2_ and 0.7% Triton X-100) and thereafter for another 3 min with the same CSK buffer supplemented with 0.3 mg/ml RNase A (CSK+R). Following these treatments, the cells were washed once again with PBS and then fixed in 4% paraformaldehyde.

### Western blotting

For western blotting, cells were harvested, washed and lysed in ice-cold lysis buffer (100 mM Tris-HCl (pH 8), 150 mM NaCl, 1% NP-40, 1% PMSF and 1% protease inhibitor cocktail) for 30 min on ice, followed by sonication. The lysates obtained were centrifuged at 14 000 r.p.m. for 15 min at 4 °C and their protein concentrations were determined with the Bradford assay (Bio-Rad, Sundbyberg, Sweden). Thereafter, western blotting was performed by to standard procedures. Cell fractionations were performed using a nuclear extraction kit according to manufacturer's instructions (Nuclear Extraction kit, Active Motif, Nordic Biolabs, Täby, Sweden).

## Figures and Tables

**Table 1:** Correlation analysis of nuclear staining for WRAP53*β versus* clinicopathological variables

**Variable**	**WRAP53β expression (% of patients)**		***P*-value**
	**Low**^a^	**High**^b^	
*Stage*			
1	14.5	3.6	0.009
2	12.3	0.7	
3	52.2	0.7	
4	15.2	0.7	

*Differentiation grade*			
High/intermediate	26.5	4.5	0.049
Low	65.5	3.5	

a Score 0–2.

b Score 3

**Table 2:** Multivariate Cox-regression analysis of histopathological parameters in relation to overall survival

**Variable**	**HR (95% CI)**	***P*-value**
*Nuclear WRAP53*β^a^		
High (score 3)	1	
Low (score 0–2)	4.20 (1.00–17.61)	0.050

*Stage*		
1	1	
2	1.91 (0.74–4.94)	
3	3.62 (1.65–7.94)	
4	10.72 (4.24–27.10)	0.000

*Differentiation grade*		
High/intermediate	1	
Low	1.06 (0.64–1.77)	0.813

*Histology*		
Mucinous	1	
Serous	0.66 (0.28–1.52)	
Endometrioid	0.45 (0.18–1.10)	
Other	0.36 (0.12–1.09)	0.147

*Levels of p53*^b^		
Negative	1	
Positive/high	1.58 (1.03–2.43)	0.036

a Data missing for six patients.

b Data missing for five patients

## Abbreviations

WRAP53: WD40-encoding RNA antisense to p53
PARP: poly ADP-ribose polymerase
HR: homologous recombination
NHEJ: non-homologous end joining
IR: ionizing irradiation
DAPI: 4′,6-diamidino-2-phenylindole
CSK: cytoskeleton buffer
siRNA: small interfering RNA
*γ*H2AX: phosphor-histone H2A.X
MDC1: mediator of DNA damage checkpoint 1
RNF8: ring finger protein 8
RNF168: ring finger protein 168
BRCA1: breast cancer 1, early onset
53BP1: tumor protein p53 binding protein 1
RAD51: RAD51 recombinase
FK2: antibody clone recognizing K^29^-, K^48^- and K^63^-linked polyubiquitinylated and monoubiquitinylated proteins.
